# Analysis of the Role of the Mc4r System in Development, Growth, and Puberty of Medaka

**DOI:** 10.3389/fendo.2019.00213

**Published:** 2019-04-05

**Authors:** Ruiqi Liu, Masato Kinoshita, Mateus C. Adolfi, Manfred Schartl

**Affiliations:** ^1^Physiological Chemistry, Biocenter, University of Wuerzburg, Wuerzburg, Germany; ^2^Division of Applied Biosciences, Graduate School of Agriculture, Kyoto University, Kyoto, Japan; ^3^Comprehensive Cancer Center Mainfranken, University Clinic Wuerzburg, Wuerzburg, Germany; ^4^Hagler Institute for Advanced Study and Department of Biology, Texas A&M University, College Station, TX, United States

**Keywords:** medaka, Mc4r, knockout, puberty, growth

## Abstract

In mammals the melanocortin 4 receptor (Mc4r) signaling system has been mainly associated with the regulation of appetite and energy homeostasis. In fish of the genus *Xiphophorus* (platyfish and swordtails) puberty onset is genetically determined by a single locus, which encodes the *mc4r*. Wild populations of *Xiphophorus* are polymorphic for early and late-maturing individuals. Copy number variation of different *mc4r* alleles is responsible for the difference in puberty onset. To answer whether this is a special adaptation of the Mc4r signaling system in the lineage of *Xiphophorus* or a more widely conserved mechanism in teleosts, we studied the role of Mc4r in reproductive biology of medaka (*Oryzias latipes*), a close relative to *Xiphophorus* and a well-established model to study gonadal development. To understand the potential role of Mc4r in medaka, we characterized the major features of the Mc4r signaling system (*mc4r, mrap2, pomc, agrp1*). In medaka, all these genes are expressed before hatching. In adults, they are mainly expressed in the brain. The transcript of the receptor accessory protein *mrap2* co-localizes with *mc4r* in the hypothalamus in adult brains indicating a conserved function of modulating Mc4r signaling. Comparing growth and puberty between wild-type and *mc4r* knockout medaka revealed that absence of Mc4r does not change puberty timing but significantly delays hatching. Embryonic development of knockout animals is retarded compared to wild-types. In conclusion, the Mc4r system in medaka is involved in regulation of growth rather than puberty.

## Introduction

Puberty is the process through which an individual develops from an immature to the mature stage and obtains first time the capability to reproduce. This process can be affected by both the genetic background and environmental factors (1, 2). Teleost fish of the genus *Xiphophorus* (platyfish and swordtails) show polymorphisms in puberty onset timing, body length and reproduction tactics (3). The *P* (*Puberty*) locus encoding the melanocortin 4 receptor (Mc4r) is critically involved in establishing these polymorphisms (4, 5). Distinct alleles of *mc4r* and copy number variation of these alleles determine the onset of puberty to be early or late (4).

Mc4r belongs to the class A of G protein-coupled receptor (GPCR) and is a member of the melanocortin receptor family, which has five members, Mc1r to Mc5r (6). These receptors regulate a wide array of functions, for example, pigmentation for Mc1r (7), and energy homeostasis for Mc4r (8). The Mc4r signaling system consists besides the receptor itself and its cognate ligands, which are processed from the agonist precursor pro-opiomelanocortin (Pomc), of the antagonist agouti-related peptide (Agrp), and melanocortin receptor accessory protein 2 (Mrap2) (6). Vertebrate Pomc is cleaved into physiological ligands α-MSH, β-MSH, γ-MSH, ACTH, but teleosts lack γ-MSH (9). All melanocortin receptors bind the four ligands, with the exception of Mc2r, which interacts exclusively with ACTH (10). Ligands have different affinities to each of the receptors (8). β-MSH has the highest affinity to Mc4r, and synthetic ligand NDP-MSH is a highly potent α-MSH analog that also activates Mc4r (11). Agrp can act as an antagonist or inverse agonist, inhibiting the activation of Mc4r by MSH or the constitutive activity of Mc4r by its N-terminus, the intramolecular ligand (12).

Mc4r has been reported to be involved in many physiological processes in several fish species. The most prominent physiological processes affected are food intake and energy balance, as reported in goldfish (13) and rainbow trout (14). In zebrafish, Mrap2 regulating Mc4r signaling controls adult somatic growth and stimulates larval growth during development (15). Zebrafish Mrap2 has two forms (16): Mrap2a is the larval form and blocks Mc4r, and Mrap2b is the adult form and enhances Mc4r function (15).

In mammals, Mc4r is mainly expressed in the central nervous system. In humans, mutations in *MC4R* are connected to early-onset obesity (17), and in mice, *Mc4r* knockout causes hyperphagic obesity (18). These mice have an obese phenotype like the *Agrp* overexpression mice (*A*^*vy*^) (19). Mc4r was shown to be involved in energy intake and expenditure and acts as a potential target for pharmacological intervention with obesity (17). Leptin and ghrelin signaling pathways are acting upstream of Mc4r, while kisspeptin is downstream of Mc4r (20). Due to its role in the center of this regulatory network, increasing the knowledge about Mc4r is instrumental for a better understanding of metabolic disorders.

Fish of the genus *Xiphophorus* provide a useful tool to study the genetic basis of puberty regulation and the molecular factors involved in this process (4, 5, 21). However, for functional studies reverse genetics cannot be applied to these fish, because they are livebearing and have internal embryo development. Medaka (*Oryzias latipes*) is a phylogenetically closely related species to *Xiphophorus*. For both species, high-quality genomes are available (22, 23), and medaka is an established genetic model to study development and physiology (24). As an egg-laying fish, comparable to zebrafish, medaka is amenable to gene knockdown, knockout and knockin (25). The embryos of medaka are transparent, and normal embryonic development has been well-described (26). However, Mc4r and its physiological function have not yet been investigated in medaka.

Although in *Xiphophorus* fish the association of distinct male polymorphisms depending on puberty timing and its regulation by Mc4r have been shown, a similar role of Mc4r in other species remains unclear. To advance our knowledge of fish puberty, we investigated the Mc4r system in medaka fish. Through analysis of the temporal and spatial expression profile and functional studies on *mc4r* mutants, we find that the role of *mc4r* is related to development and growth in medaka, like in zebrafish, but not to controlling puberty timing like in *Xiphophorus* fish.

## Materials and Methods

### Animals

All wild-type medaka used in the study were from the Carbio strain. The knockout *mc4r* medaka mutant “−2/+3” was generated by TALEN technology. For details of the generation of the mutant see (27). Adult medaka (*Oryzias latipes*) were maintained under a standard light/dark cycle of 14/10 h at 26°C in the fish facility of Biocenter at the University of Wuerzburg. Eggs of medaka fish were collected and cultured in Danieau's medium (NaCl 17.4 mM, KCl 0.21 mM, MgSO_4_ 0.12 mM, Ca(NO_3_)_2_ 0.18 mM, HEPES 1.5 mM, methylene blue 0.0001%) until hatching.

All animals were kept and sampled in accordance with the applicable EU and national German legislation governing animal experimentation, in particular, all experimental protocols were approved through an authorization (568/300–1,870/13) of the Veterinary Office of the District Government of Lower Franconia, Germany, in accordance with the German Animal Protection Law (TierSchG).

### Phylogenetic Analysis

The annotated Mc4r, Mrap2, Pomc, Agrp1 sequences from human and 14 fish species were retrieved from either National Center for Biotechnology Information nucleotide sequences database (NCBI)^1^ or Ensembl genome browser^2^. Protein sequences were used in the phylogenetic analysis and were aligned by ClustalW from the software package MEGA7 (28). Evolutionary analyses were conducted with MEGA7 based on maximum likelihood method with 1,000 bootstrap replicates.

Eleven Actinopterygians (ray-finned fish) including Amazon molly (*Poecilia formosa*), cave fish (*Astyanax mexicanus*), cod (*Gadus morhua*), fugu (*Takifugu rubripres*), medaka (*Oryzias latipes*), Southern platyfish (*Xiphophorus maculatus*), spotted gar (*Lepisosteus oculatus*), stickleback (*Gasterosteus aculeatus*), tetraodon (*Tetraodon nigroviridis*), tilapia (*Oreochromis niloticus*), zebrafish (*Danio rerio*); one Sarcopterygian (lobe-finned fish), coelacanth (*Latimeria chalumnae*); one Chondrichthyan (cartilaginous fish), elephant shark (*Callorhinchus milii*); and one Agnathan (jawless fish), lamprey (*Petromyzon marinus*) were used in the study. Common carp (*Cyprinus carpio*) was additionally used in Mrap2 phylogeny. These were compared with human (*Homo sapiens*). Accession numbers are listed in Table S1.

### Quantitative Gene Expression Analysis

To determine the time course of expression of genes from the Mc4r signaling system, three pools of medaka embryos and larvae from 0, 1, 2, 3, 4, 5, 6, 8, 10, 15, 20 days post fertilization (dpf) were collected (0 dpf *n* = 100, 1–4 dpf *n* = 50, 5–8 dpf *n* = 30, 10–20 dpf *n* = 15). The exact amounts of transcripts could not be determined in 0 dpf sample due to lack of proper normalization factors. To determine the tissue expression of Mc4r signaling pathway genes, three pools of male or female adult fish tissues (*n* = 3–4) of brain, eye, gill, kidney, liver, gonad, skin, and muscle were analyzed. Total RNA was isolated using TRIZOL reagent (life technologies, Carlsbad, California, United States). After DNase treatment, a reverse transcriptase with random hexamer primers was used to synthesize first strand cDNA using RevertAid First Strand cDNA Synthesis Kit (Thermo Scientific, Waltham, Massachusetts, United States) according to manufacturer's manual. Primers for *mc4r, mrap2, pomca, pomcb, agrp1*, and housekeeping gene *ef1a* are listed in Table S2. cDNA was applied to Reverse Transcription Quantitative PCR (RT-qPCR) using SYBR Green reagent on a Mastercycler realplex^2^ Eppendorf machine Eppendorf (Eppendorf, Hamburg, Germany). Data were analyzed by the ΔΔCT method and normalized to housekeeping gene *ef1a*. The values were presented as mean ± standard deviation (SD).

### Whole Mount *in situ* Hybridization

The spatial expression patterns of *mc4r* and *mrap2* were analyzed by whole mount RNA *in situ* hybridization following procedures described previously (29–31). To generate riboprobes, cDNA from brain were used to amplify both genes (primers listed in Table S2). The amplicons were cloned into a pGEM-T Easy vector and verified by sequencing. Plasmids were linearized, and *in vitro* transcription was performed by T7 or SP6 RNA polymerase (Roche, Basel, Switzerland) with digoxigenin or fluorescein RNA labeling mix (Roche). Whole mount *in situ* hybridization was performed on dissected intact brains fixed in 4% PFA and dehydrated in methanol for storage. Before hybridization brains were rehydrated with PBST and digested with 10 μg/ml proteinase K for 45 min. Samples were hybridized with riboprobes overnight at 68°C in a humidified chamber and then were subjected to stringent washes of SSC series (0.05X SSC used for washes of high stringency) and by PBST. The samples were embedded in 3% agarose and 100 μm section were cut by TPI Vibratome Series 1000 Sectioning System (Technical Products International Inc., St. Louis, Missouri, United States). After blocking with 5% sheep serum, anti-digoxigenin-AP (1:5,000) or anti-fluorescein-AP (1:2,000) were applied to the sections overnight at 4°C. NBT/BCIP solution (Roche) or FastRed Tablets (Sigma, St. Louis, Missouri, United States) were used to develop the signals by AP reaction according to manufacturer's instruction. Double *in situ* hybridization was performed by first visualizing fluorescein probes and thereafter digoxigenin probes. Sections were mounted in 80% glycerol for microscopy (Zeiss AxioPhot, using AxioVision Rel 4.8 software, Oberkochen, Germany). Fluorescence pictures were taken with a Confocal microscope (Nikon Eclipse Ti C1, using NIS-Elements AR software, Minato, Tokyo, Japan). The transcript localization was determined according to the medaka brain atlas (32).

### Functional Analysis of Knockout Fish

Medaka wild-type (WT) fish (Carbio strain) and Mc4r knockout (KO) −2/+3 TALEN-KO strain (27) (*mc4r* wt and mutant sequences provided in Supplementary Data) were compared with respect to hatching time, puberty onset and growth. Three groups of 60–200 wild-type and knockout embryos were collected, and the hatching time of each embryo was recorded. Embryo development from day 0 to day 7 was compared. Linear growth (forehead to trunk terminus) of larvae was imaged by stereomicroscope (Nikon SMZ1000 microscope, Minato, Tokyo, Japan, with LEICA DFC450C to capture images, using LAS V4.1 software, Wetzlar, Germany) and measured using ImageJ 1.51 (33, 34).

Three groups of 25 wild-type and 22–25 knock-out fish were raised in a two-chamber aquarium (Figure S3B) to guarantee identical rearing conditions for two groups. Puberty refers to the stage when fish become sexually mature and acquire for the first time the ability to reproduce (1). Since medaka has obvious secondary sexual characters and a special courtship behavior during the mating process, here puberty onset was determined for females as the day of the first eggs release and for males by the appearance of papillary processes in the anal fin (35). Three independent experiments were conducted. The linear growth of juveniles and adult fish were measured by rulers. The growth of juveniles (the period when males and females are undistinguishable) was measured and compared between KO and WT. The linear length and the age of adults were measured at the time of puberty, and KO and WT fish were compared.

Statistical treatment of the data was done with GraphPad Prism 6 software. Two-way *ANOVA* was used for comparison of WT and KO females and males for the time of puberty onset and the size at puberty. To compare each sample with every other sample, corrections were made for multiple comparisons by Sidak's test. Multiple *t*-test with correction for multiple comparison by Holm-Sidak method was used to compare the WT and KO growth curves. One-way *ANOVA* was used for comparison of larval length and to compare KO day7 and KO hatch with WT hatch, corrections were made for multiple comparisons by Dunnett's test. The values were presented as the mean ± SD. Mann-Whitney test was used to compare WT and KO hatching time. Data are presented in box plots; whiskers are from min to max. In Kaplan-Meier plots for puberty, data were compared with the log-rank (Mantel-Cox) tests. GraphPad Prism 6 was used to display the results.

## Results

### Mc4r Signaling System Genes in Fish

The phylogenetic trees of the Mc4r signaling system genes show in general a topology that follows the known organismal relationships (Figure S1A). Interestingly, *mc4r* of Southern platyfish and blind cavefish have longer branches indicating faster evolution toward specialized functions in these lineages. Different from all other species, only zebrafish has two copies of *mrap2* (Figure S1B) which have previously been assigned to different functions for growth regulation (15). Thus, this appears to be a special situation in zebrafish, which cannot be generalized. However, search in common carp (*Cyprinus carpio*) genome reveals also two copies of *mrap2, mrap2a*, and *mrap2b* (Table S1). Two *pomca* and one *pomcb* sequences were found in several teleosts, including Southern platyfish and medaka (Table S1). More basal teleost groups, on the other hand, like zebrafish and cavefish have only one *pomca*. The *agrp1* gene is present as single copy gene in all analyzed ray-finned fish including medaka (Figure S1C).

An important feature of *mc4r* as a puberty gene determining a sexually selected trait in *Xiphophorus* is sex-chromosome linkage. It is located in Southern platyfish on linkage group (LG) 21, which is the sex chromosome. However, in medaka, *mc4r* is on linkage group LG 20 and not on the sex chromosome (LG 1). None of the other Mc4r signaling system genes are sex-linked, neither in *Xiphophorus* nor in medaka (Table S3).

### Developmental and Organ-Specific Expression of Mc4r Signaling System Genes

**Figure 1:**
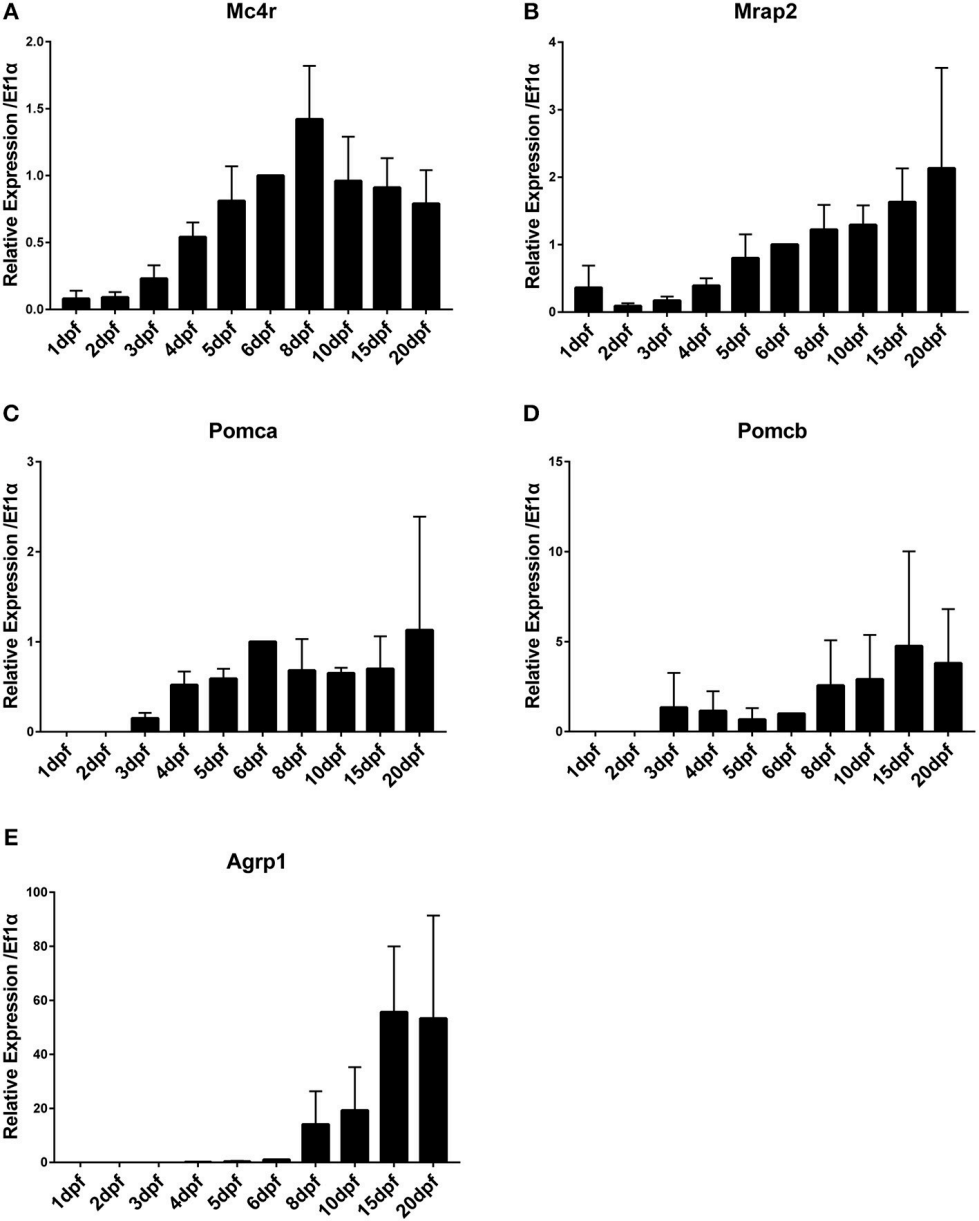
Time course of gene expression during medaka embryonic and larval development. **(A)** Mc4r, **(B)** Mrap2, **(C)** Pomca, **(D)** Pomcb, **(E)** Agrp1. Expression levels are normalized to *ef1a* and relative to expression observed in 6 dpf. Relative expression levels are presented as mean ± SD.

Analysis of the temporal expression pattern of *mc4r* signaling system genes during embryonic development revealed presence of transcripts of *mc4r* and *mrap2* already at 0 dpf (stage 10–11 blastula stage, developmental stages see Table S4), indicating that these are maternal mRNA contribution (data not shown). Especially for *mrap2*, the maternal contribution appears to be very high. Other genes are expressed exclusively from zygotic transcription. Expression of *mc4r* increases gradually starting from 1 dpf and stabilizes around 5 dpf (Figure 1A). Expression of *mrap2* increases gradually from 1 dpf on (Figure 1B). Expression of *pomca* starts and increases from 3 dpf on (Figure 1C). Expression of *pomcb* initiates at 3 dpf at low levels and increases at 6–8 dpf (Figure 1D). Expression of *agrp1* starts with very low levels at 4–5 dpf and is highly upregulated at 8 dpf (Figure 1E). Expression of *agrp1* shows a dramatic upregulation after the fish start to feed.

**Figure 2:**
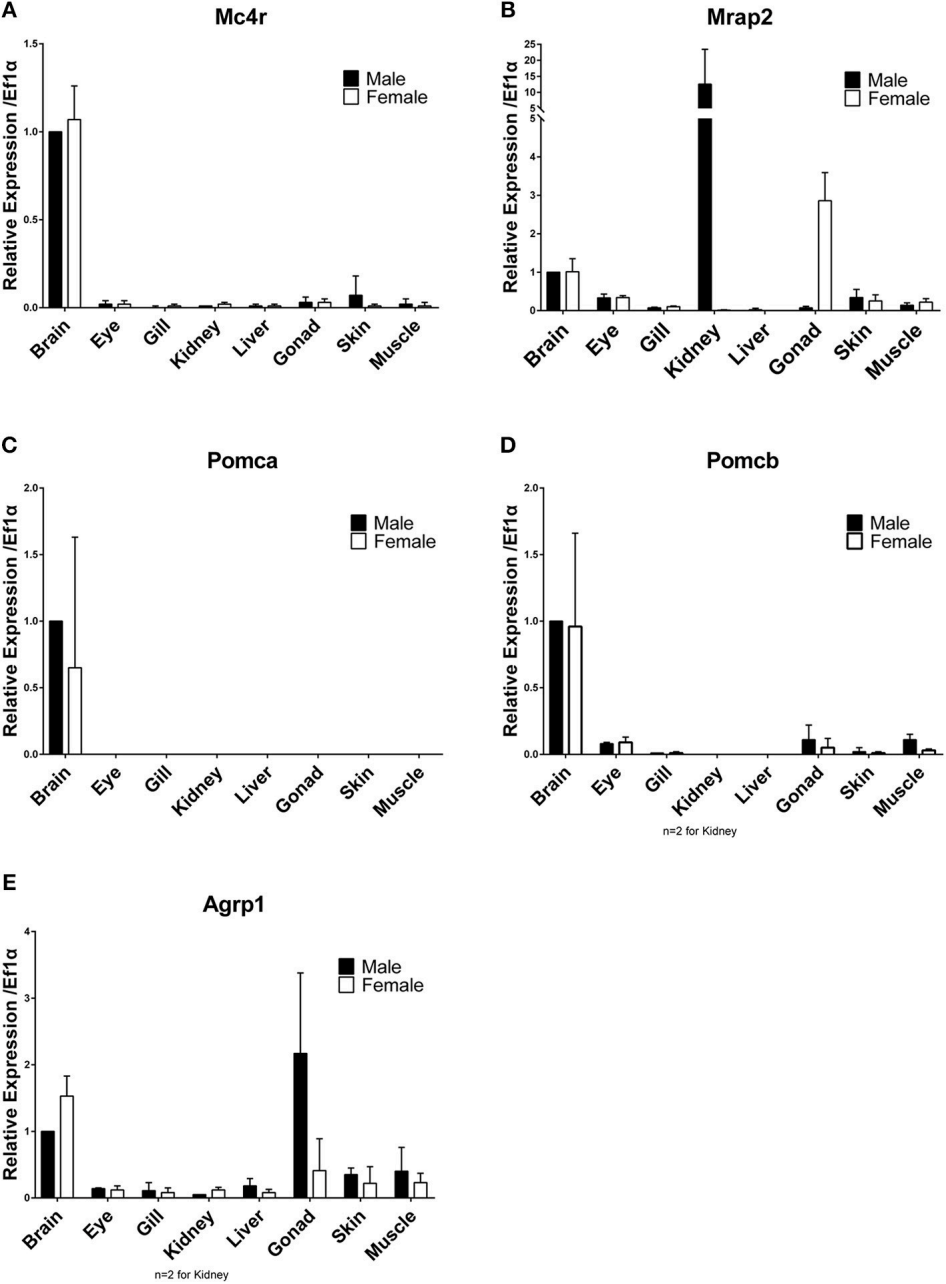
Differential tissues gene expression in adult males and females. **(A)** Mc4r, **(B)** Mrap2, **(C)** Pomca, **(D)** Pomcb, **(E)** Agrp1. Expression levels are normalized to the expression level of *ef1a* and relative to expression observed in male brains. Relative expression levels are presented as mean ± SD.

Differential expression among tissues and between males and females were analyzed using RT-qPCR. Brains of both sexes highly express *mc4r* and *mrap2* (Figures 2A,B). High expression of *mc4r* in brains of medaka confirms previous analysis (5). The *pomca, pomcb* and *agrp1* genes were also highly expressed in brains of both sexes (Figures 2C–E). The *agrp1* gene has a higher (1.5-fold) expression in the female brain (Figure 2E). Besides brain, *mc4r* is also expressed in all tested tissues at low levels; except for skin, all the rest are background expression (Figure 2A). The *mrap2* gene shows remarkably high expression in male kidney (12.6-fold higher than brain) and ovary (2.9-fold) (Figure 2B). No other tissues express *pomca*, but *pomcb* showed low expression in eyes, gills, gonads, skins and muscles (Figures 2C,D). Testis expresses high levels of *agrp1*, while there is only low expression in all other tissues (Figure 2E). In summary, high expression of all *mc4r* signaling system genes during ontogenesis when the larvae start to feed and in the brain of adult fish is in agreement with functions in appetite regulation and energy homeostasis in medaka. The sexually dimorphic expression pattern of *mrap2* (kidney, gonad) and *agrp1* is intriguing and warrants further studies to elucidate a sex-related function.

### Co-localization of *mc4r* and *mrap2* in the Adult Brain

Mrap2 has been described as Mc4r accessory protein of important function (15). To exert such function *mrap2* has to be co-expressed with *mc4r* in the same cells. To investigate the localization of *mc4r* and *mrap2* expression, whole mount *in situ* hybridization was performed using intact brains from adult females and males.

**Figure 3:**
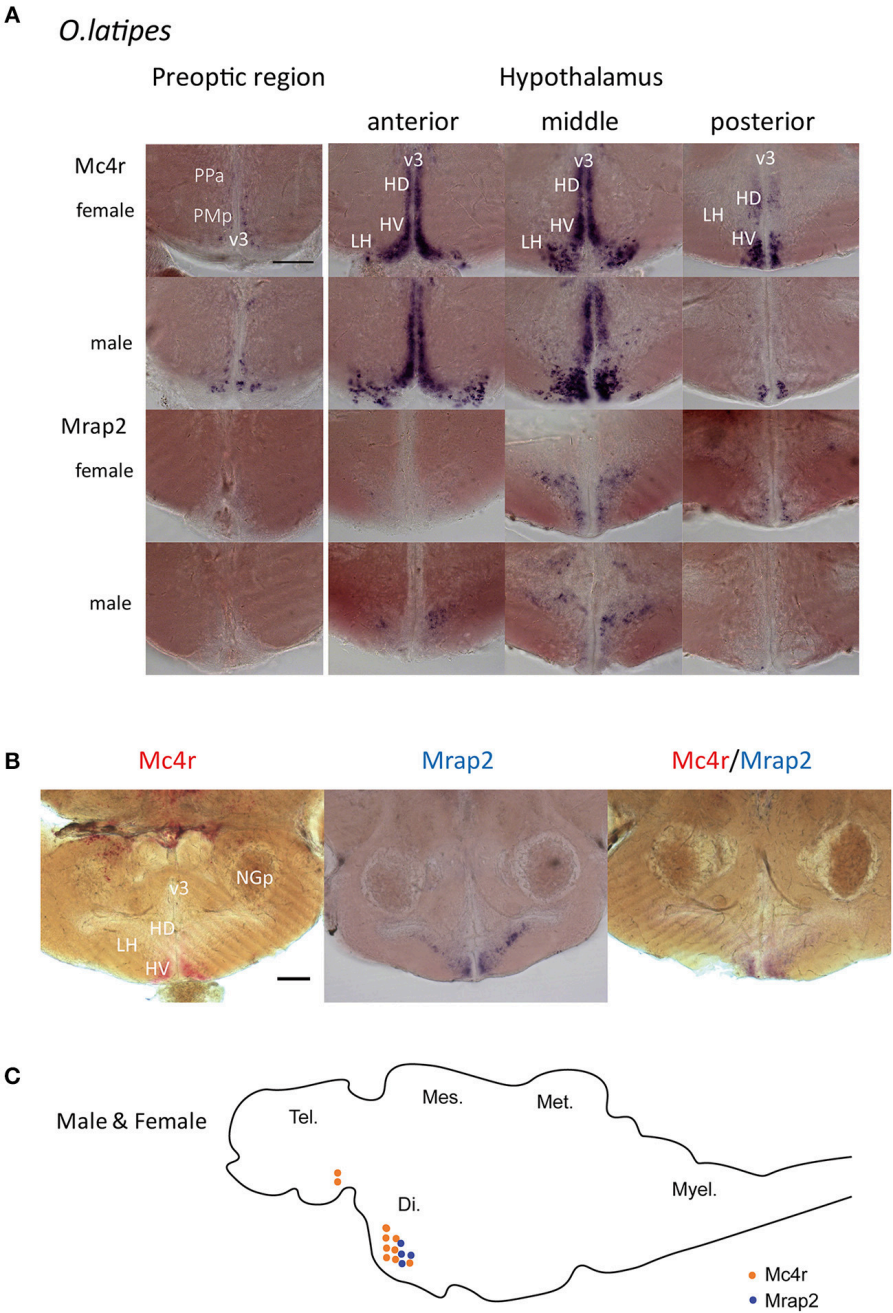
Whole mount *in situ* hybridization for mRNA localization of *mc4r and mrap2* in adult male and female brains. **(A)** Expression localization of *mc4r* in the preoptic region and in the hypothalamus, and of *mrap2* in the medial hypothalamic region. Both genes presented the same expression pattern for males and females. Scale bar: 100 μm. **(B)** Double *in situ* hybridization in the hypothalamic region confirms the co-localization of *mc4r* (red) and *mrap2* (purple). Scale bar: 100 μm. **(C)** Schematic representation of *mc4r* (orange) and *mrap2* (blue) mRNA localization. Brain regions: Di., diencephalon; Tel., telencephalon; Mes., mesencephalon; Met., metencephalon; Myel., myelencephalon. Brain areas in detail: v3, third ventricle; PPa, anterior parvocellular preoptic nucleus; PMp, parvocellular part, magnocellular preoptic nucleus; HD, dorsal periventricular hypothalamus; HV, ventral periventricular hypothalamus; LH, lateral nucleus of hypothalamus; NGp, posterior glomerular nucleus.

While *mc4r* was expressed in the preoptic region and hypothalamus region, *mrap2* was only expressed in the hypothalamus (Figure 3A; Figure S2A). In the hypothalamus, *mc4r* was expressed more anteriorly than *mrap2*. In addition, the expression region of the two genes was also overlapping in the central part of the hypothalamus (Figure 3B; Figure S2B). The anatomical overlap of the expression domains may indicate that both genes are co-expressed on the cellular level. There was no noticeable difference between males and females (Figure 3C). The fact that only a subset of *mc4r* expression domains overlap with that of *mrap2* indicates that in certain areas of the brain the Mc4r signaling system may function without the accessory protein.

### Effect of Mc4r Knockout on Hatching Timing and Linear Growth

**Figure 4:**
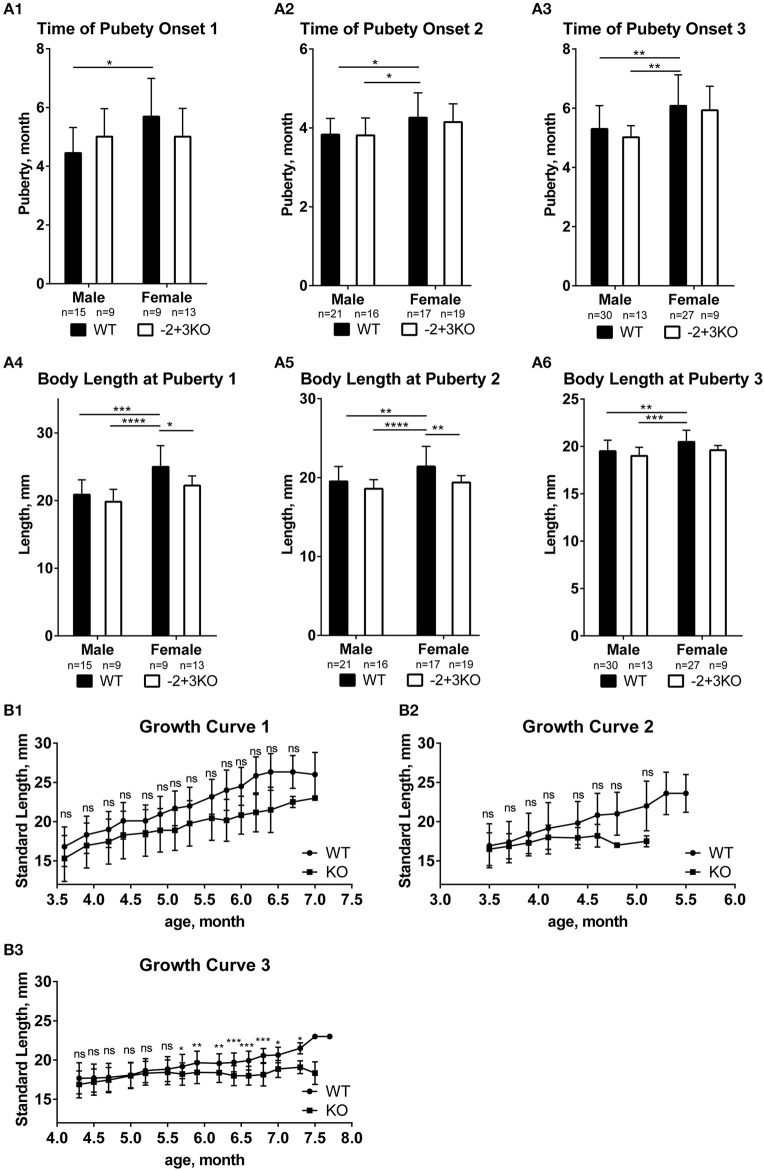
Effect of *mc4r* knockout on medaka growth and puberty. **(A)** No differences in puberty onset timing in the three trials **(A1, A2, A3)**, but some differences in body length at puberty between WT and KO medaka females and males in three replicates **(A4, A5, A6)**. Data are presented as mean ± SD. Statistical significance after two-way *ANOVA* is indicated as asterisks (*). **(B)** The standard length of WT and KO during growth in the three experiments **(B1, B2, B3)**. Data are presented as mean±SD. Statistical significance after multiple *t*-test is indicated as asterisks (*). For all statistics: ns: not significant, *p < 0.05, **p < 0.01, ***p < 0.001, ****p < 0.0001.

To investigate if *mc4r* would have a similar function as puberty onset determining gene in medaka like *Xiphophorus* fish, puberty onset was monitored in a medaka *mc4r* knockout line (27). We used a line that has the −2/+3 mutation (*mc4r* sequences in Figure S3A, Supplementary Data, these indels in the *mc4r* gene were verified by genotyping and sequencing) and compared to wild-type medaka. In medaka, puberty onset can be determined from the development of secondary sexual characteristics in males (anal fin papillary processes), which can be easily observed under a stereomicroscope. In females, the first egg-laying indicates puberty completion (36). We found no significant difference in puberty timing between KO and WT, neither in male fish nor in female fish (Figure 4A; Figure S3C). Body length at puberty also did not differ between KO and WT in males. However, KO females matured at shorter body length than WT female (Figure 4A). Moreover, the average standard body length of the KO fish was generally shorter than that of WT although the difference is not significant, except at some later time points in trial 3 (Figure 4B), showing that the knock-out fish have a trend of a slower linear growth rate.

**Figure 5:**
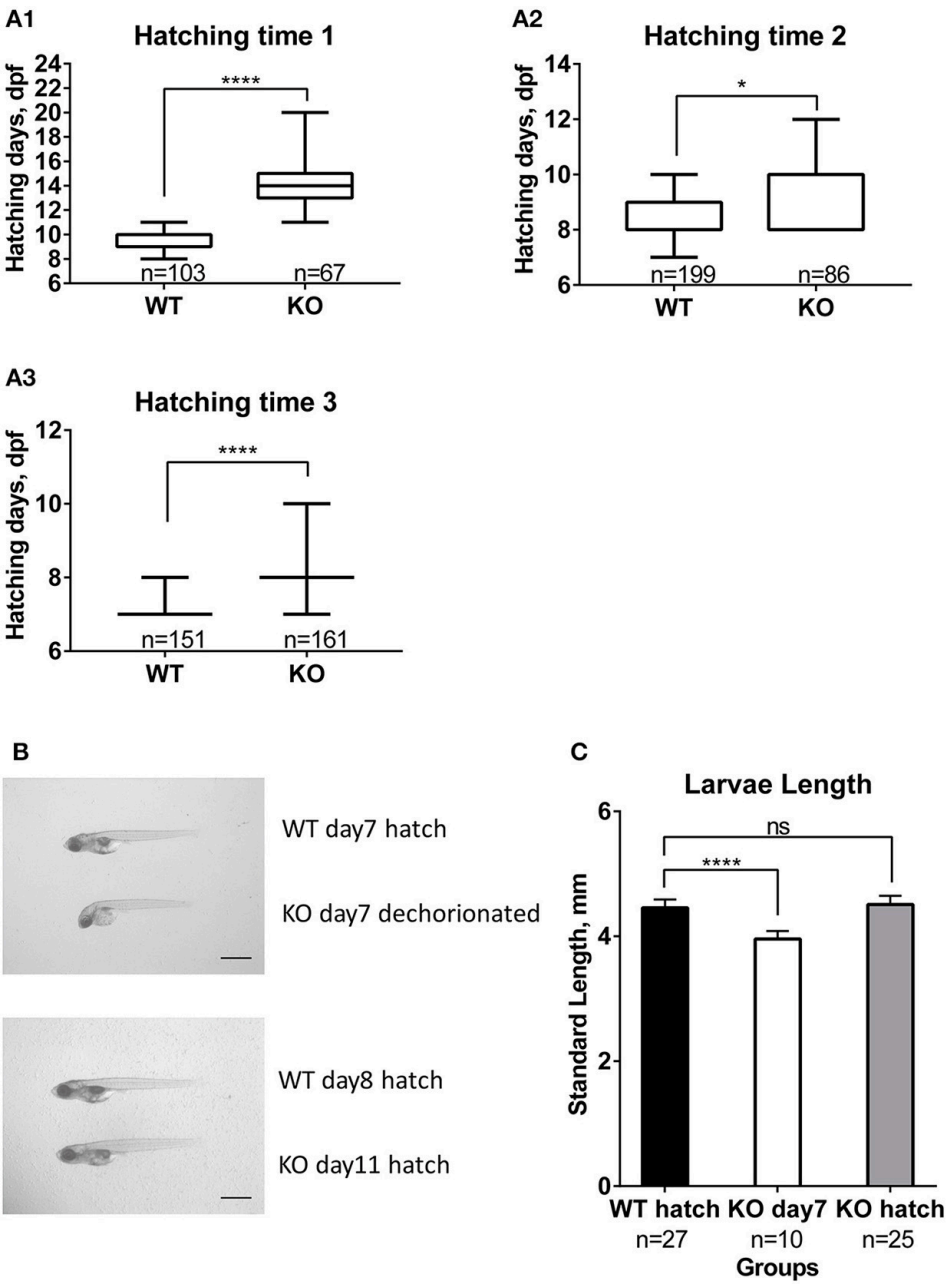
Effect of *mc4r* knockout on medaka development and hatching. **(A)** Differences in hatching time of WT and KO medaka in the three trials **(A1, A2, A3)**. Data are presented in box plots; whiskers are from min to max. Statistical significance after Mann-Whitney test is indicated as asterisks (*). **(B)** Photographs of larvae at 7 dpf and at hatch for WT and KO. Scale bar: 1 mm. **(C)** Graphic showing slower development of KO larvae compared to WT, apparent by a shorter length at 7 dpf. Data are presented as mean±SD. Statistical significance after one-way *ANOVA* is indicated as asterisks (*). For all statistics: ns: not significant, *p < 0.05, ****p < 0.0001.

We also noticed that hatching time was delayed for KO fish. Compared to zebrafish, medaka fish develop slower and stay in the chorion until much more advanced developmental stages (26, 37). This allowed us to better distinguish changes in the timing of hatching. From three independent experiments, the KO fish clearly showed significantly delayed hatching, for at least 1 day (Figure 5A). In order to observe if this is due to a possible change in embryo development and growth, we followed the development of WT and KO embryos. Starting at day one the KO embryos were about one stage slower than WT (data not shown). The differences became more obvious for embryos from day three on, where the body of KO fish was covering smaller portions of the yolk sphere than of WT fish. When we compared the standard body length of freshly hatched WT (day 7) and KO fish dechorionated on day 7, KO larvae were significantly shorter than WT (Figures 5B,C). Finally, we compared the body length of freshly hatched KO to WT. The hatched KO had the same size as hatched WT (Figures 5B,C) despite being 1–4 days older. All in all, these results indicate that *mc4r* in medaka is more involved in regulating growth than the onset of puberty.

## Discussion

### Characterization of the Mc4r Signaling System in Medaka

As the first step toward an investigation of the Mc4r signaling system genes in medaka fish, we performed an evolutionary analysis of Mc4r signaling system genes in various fish. Contrary to Southern platyfish, in which *mc4r* is present in multiple copies on the sex chromosome (4, 5), the medaka *mc4r* is a single copy gene. This confirms now on the whole genome sequence level the earlier evidence from Southern blot data (5). Moreover, the medaka gene is not located on the sex chromosome. In the Mc4r phylogeny, the long branch for the Southern platyfish indicates considerable protein sequence divergence which we hypothesize as being due to special functions of Mc4r in regulating puberty onset that evolved in the *Xiphophorus* fish lineage (4, 5). Similarly, the long branch for cavefish reflects a special adaptation of Mc4r, e.g., in control of energy metabolism and adaptation to limited nutrients in the caves (38).

The *mrap2* gene has been reported so far to have two copies only in zebrafish (15). We found also two copies in common carp. This indicates a lineage-specific duplication in cyprinids. The two *pomc* paralogs (*pomca* and *pomcb*) probably arose during the 3rd round of whole genome duplication of the teleost, since only one copy of *pomc* is present in other vertebrates. However, two sequences of *pomca* (*pomca1* and *pomca2*) were found in most of Percomorpha teleost, indicating a lineage-specific duplication of this gene. The *agrp* gene has been found in some species to exist in two versions, *agrp1* and *agrp2*. *agrp1* (also named *agrp* previously) has been related to obesity (39, 40) and was described as an antagonist and inverse agonist of Mc4r. On the other hand, *agrp2* [in some fish named as *asip2b* (41, 42)] is involved in background adaptation (43) and pigment pattern formation (44). Taken together, on the *mc4r* signaling system gene level, the *Xiphophorus* fish and medaka are similar.

In the next step toward a physiological characterization of the Mc4r signaling system of medaka, temporal expression analysis showed mRNA maternal contribution at low levels for *mc4r* and high levels for *mrap2*. The *mc4r* and *mrap2* genes, which code for membrane proteins, are expressed early in development, concurrent with the late neurula stage and early brain development. Thus, *mc4r* and *mrap2* genes are expressed already in early and late embryonic stages, when the brain and integrated functional proteins in the neurons are developing. The *pomc* genes, precursors of Mc4r agonists, start expression 2 days after *mc4r* and *mrap2*. At the hatching stage, all genes are expressed, indicating that the whole system is in place for full function when the fish start feeding. Intriguingly, *agrp1* showed a dramatic upregulation after hatching, coinciding with first feeding. This may be explained by the important role of *agrp1* in appetite regulation and implies the role of the Mc4r system in energy metabolism regulation from the very first feeding event (45, 46).

Spatial expression analysis revealed that the *mc4r* signaling pathway genes are expressed highly in brains of both sexes consistent with the center of food uptake regulation in the hypothalamus and neighboring regions of the brain, e.g., the preoptic area (46). The expression domains of *mc4r* and *mrap2* were identified in the hypothalamus, and the two genes showed colocalization in certain areas. This indicates interaction of Mc4r and Mrap2 in those cells. Mc4r and Mrap2 are both membrane proteins, their interaction could tune signal strength and intracellular signal transduction as previously proposed for the zebrafish homologs (47). At some locations where *mc4r* is expressed, *mrap2* is not present, implying that in such regions *mc4r* may be able to transmit signals without its co-factor *mrap2*. Nevertheless, *mrap2* expression was observed only in regions where the *mc4r* signal was present, supporting the specific role of Mrap2 in interacting with Mc4r in the brain (15).

The high transcript levels of *mrap2* in ovary could be explained by the very high maternal RNA contribution. Mature eggs contain high levels of *mrap2* inferred from maternal contribution level, and this is reflected in the high mRNA levels in the ovary. High expression of *mrap2* in the male kidney has been shown before. In zebrafish, *mrap2* is highly expressed in the head kidney (47). Moreover, *mrap2* is expressed mainly in brain and adrenal gland in human (48) and widely expressed including kidney in mice (49). Fish *mrap2* might be similarly expressed in the interrenal gland on the kidney. A male-specific function of *mrap2* in kidney still needs to be investigated.

### Effect of Mc4r on Puberty Timing and Adult Growth

The expression data did not exclude that the Mc4r signaling system in medaka could have the same function in regulating puberty timing as in *Xiphophorus* fish. In *Xiphophorus, mc4r* is expressed highest in the brain (5). We find also in adult medaka that the Mc4r signaling system genes are expressed mainly in the preoptic and hypothalamic region, similar as in zebrafish (47). However, our analysis of the Mc4r KO fish revealed that males and females of the WT and Mc4r KO strain reach puberty at a similar time. However, WT females reach puberty significantly later than WT males, while KO females and males do not show a significant difference on puberty timing. In zebrafish, because of the difficulty to monitor puberty onset in this species (50), an effect of *mc4r* on puberty timing was not noted. Our results indicate that Mc4r may not be the critical puberty signal in medaka fish. Alternatively, the lack of a puberty phenotype in our TALEN KO fish might be due to the fact that other genes can compensate for the loss of Mc4r function (51).

Although the timing of puberty onset is not altered in KO fish, female body length at puberty in KO fish is significantly shorter than WT. WT Females are significantly larger than WT males and KO males. KO females and males do not show a difference in body length at puberty. Since puberty is normally related to animal size/weight, earlier puberty and shorter length at puberty in KO females implies an advanced pubertal process in KO females. This suggests that the Mc4r function is relevant for female growth in medaka, rather than male growth. This is unlike the situation in Southern platyfish and other *Xiphophorus* species, where Mc4r is involved in male size but has no noticeable effect on female growth and adult size. In addition, KO fish are slightly shorter than WT, indicating that possibly growth in general is retarded in Mc4r KO fish. In zebrafish, *mc4r* KO led to increased body length at 42 dpf (52). Overexpression of the Mc4r inverse agonists, both Agrp1 and Asip1, increased linear growth in zebrafish (53, 54). *mc4r* KO in medaka surprisingly show retarded growth suggesting that eventually other receptors like Mc3r, which is also related to energy balance and reacts with Pomc and Agrp1, could compensate for the function of compromised Mc4r by responding to Pomc signals and thus reducing growth.

### A Role for Mc4r in Medaka Embryonic Development and Larval Growth

Intriguingly, we observed that the hatching time of Mc4r KO medaka was affected. The Mc4r KO fish show delayed development from day one coinciding with the onset of *mc4r* expression. This effect becomes even more obvious from day three on. It could be speculated that Mc4r has a role in medaka development by influencing growth. Zebrafish Mc4r KO, however, does not show changes in linear growth during embryonic and larval stages. An increased length of Mc4r KO zebrafish was first seen only at 42 dpf (52). The embryogenesis of zebrafish is fast and the fish hatch already at day 2–3, compared to day 7–9 in medaka. Thus, a delay in zebrafish development may have remained undetectable. The overall growth of medaka was clearly slowed down in KO larvae. It should be noted that the knock-out of Mc4r in medaka decreases growth rate, rather than increasing the body length. This is another difference to *Xiphophorus* fish (4), where the non-functional mutant Mc4r leads to increased body size. Also, in zebrafish, the loss of *mc4r* led to increased body length at 42 dpf (52).

In summary, the regulatory network of Mc4r signaling system in medaka appears not to be involved in puberty regulation as in *Xiphophorus*, but rather in growth and development. Our findings suggest that from an evolutionary perspective the role of *mc4r* as the critical *P* locus gene is a specific innovation in the *Xiphophorus* lineage.

## Author Contributions

RL performed the phylogeny, sampling, RT-qPCR, *in situ* hybridizations, KO adult and embryos functional analysis, and drafted the manuscript. MK established and provided the KO medaka line. MA supervised the experiments and helped to review the manuscript. MS defined and designed the study, coordinated all steps of the research and reviewed all versions of the manuscript.

### Conflict of Interest Statement

The authors declare that the research was conducted in the absence of any commercial or financial relationships that could be construed as a potential conflict of interest.

## Footnotes

^1^https://www.ncbi.nlm.nih.gov/

^2^http://www.ensembl.org/index.html

## Abbreviations

Agrp: agouti-related peptide
dpf: days post fertilization
GPCR: G protein-coupled receptor
KO: knockout
LG: linkage group
Mc4r: melanocortin 4 receptor
Mrap2: melanocortin receptor accessory protein 2
Pomc: pro-opiomelanocortin
SD: standard deviation
WT: wild-type.
