# Supplementary material for: Analysis of the Role of the Mc4r System in Development, Growth, and Puberty of Medaka
Source: Front Endocrinol (Lausanne). 2019 Apr 5;10:213. doi: 10.3389/fendo.2019.00213 (PMC6463759; doi:10.3389/fendo.2019.00213)
Supplement: Supplementary file 1 [file Data_Sheet_1.pdf]

Frontiers in Endocrinology, Volume 10

Supplementary Information

Analysis of the Role of the Mc4r System in Development, Growth, and Puberty of Medaka

Ruiqi Liu, Masato Kinoshita, Mateus C. Adolfi, Manfred Schartl

Supplementary Material

Figure legends

Figure S1. Phylogenetic analysis of fish species. **(A)** Mc4r (Mc5r as outgroup), **(B)** Mrap2 (Mrap1 as outgroup), **(C)** Agrp1 (Agrp2 as outgroup). The phylogeny was inferred using the maximum-likelihood method. The percentage of replicate trees in which the associated taxa clustered together in the bootstrap test (1000 replicates) are shown next to the branches. Branches with bootstrap lower than 60 were collapsed.

Figure S2. Whole mount *in situ* hybridization detection of *mc4r* and *mrap2* genes in adult brains. **(A)** *mc4r* and *mrap2* sense probes used as negative control showing no background signals in male and female brains. Scale bar: 100 µm. **(B)** *mc4r* and *mrap2* were in part co-expressed in the same region of the hypothalamus. Scale bar: whole section 200 µm, magnified view 100 µm. Brain areas in detail: v3: third ventricle, PPa: anterior parvocellular preoptic nucleus, PMp: parvocellular part magnocellular preoptic nucleus, HD: dorsal periventricular hypothalamus, HV: ventral periventricular hypothalamus, LH: lateral nucleus of hypothalamus, OT: optic tectum, PGZ: periventricular grey zone, vm: mesencephalic ventricle, NDTL: diffuse nucleus of lateral torus, Tl: longitudinal torus.

Figure S3. Effect of *mc4r* on medaka development and puberty. **(A)** Mc4r knockout is a -2+3KO, which has a deletion of 2 nt and an addition of 3 nt at the TALEN cut site. This creates a frameshift mutation, resulting in a truncated protein. **(B)** Schematic drawing of the two-chamber aquarium, which warrants an identical environment for WT and KO. **(C)** Percentage of fish reaching puberty at a certain age. Note: males and females show no significant difference in puberty timing in three trials (males: **C1, C3, C5**; females: **C2, C4, C6**).

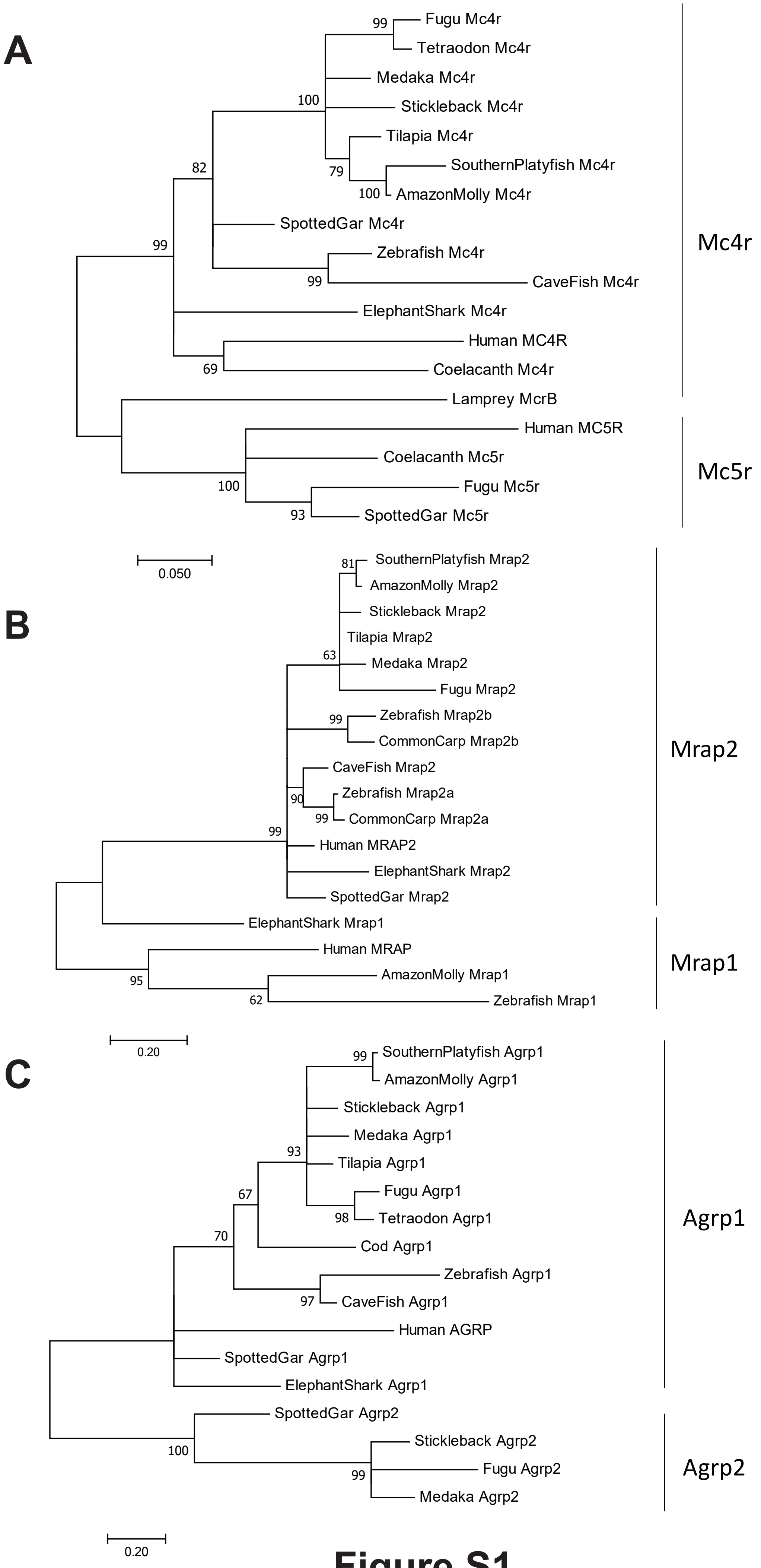
A
99
Fugu Mc4r
Tetraodon Mc4r
Medaka Mc4r
100
Stickleback Mc4r
Tilapia Mc4r
SouthernPlatyfish Mc4r
79
100
AmazonMolly Mc4r
82
SpottedGar Mc4r
99
Zebrafish Mc4r
99
CaveFish Mc4r
ElephantShark Mc4r
Human MC4R
69
Coelacanth Mc4r
Lamprey McrB
Human MC5R
Coelacanth Mc5r
100
Fugu Mc5r
93
SpottedGar Mc5r
Mc4r
Mc5r
0.050
B
81
SouthernPlatyfish Mrap2
AmazonMolly Mrap2
Stickleback Mrap2
Tilapia Mrap2
63
Medaka Mrap2
Fugu Mrap2
99
Zebrafish Mrap2b
CommonCarp Mrap2b
CaveFish Mrap2
Zebrafish Mrap2a
90
99
CommonCarp Mrap2a
99
Human MRAP2
ElephantShark Mrap2
SpottedGar Mrap2
ElephantShark Mrap1
Human MRAP
95
AmazonMolly Mrap1
62
Zebrafish Mrap1
Mrap2
Mrap1
0.20
C
99
SouthernPlatyfish Agrp1
AmazonMolly Agrp1
Stickleback Agrp1
Medaka Agrp1
93
Tilapia Agrp1
67
Fugu Agrp1
98
Tetraodon Agrp1
70
Cod Agrp1
Zebrafish Agrp1
97
CaveFish Agrp1
Human AGRP
SpottedGar Agrp1
ElephantShark Agrp1
SpottedGar Agrp2
Stickleback Agrp2
100
Fugu Agrp2
99
Medaka Agrp2
Agrp1
Agrp2
0.20

Figure S1

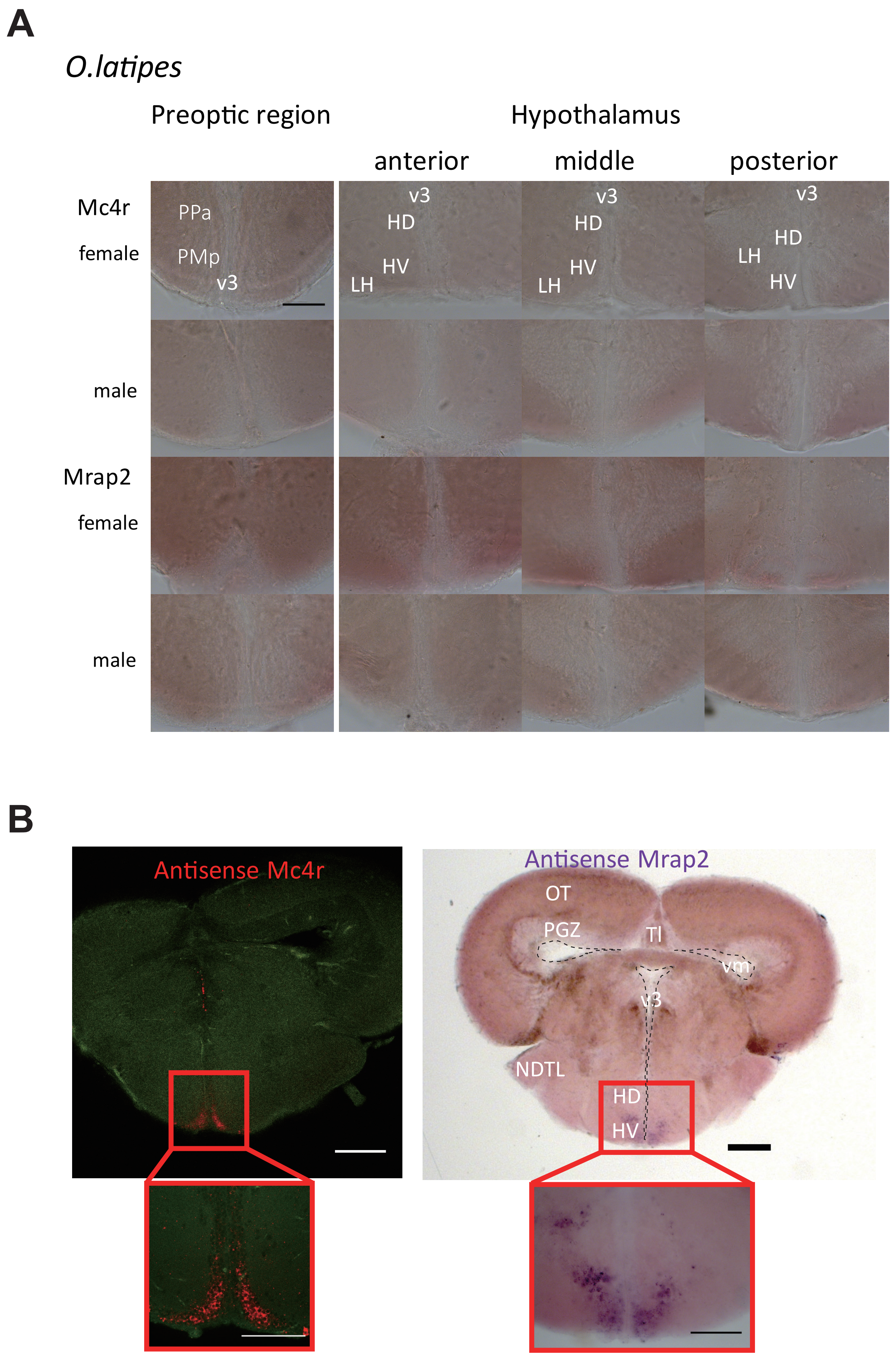

A
O.latipes
Preoptic region
Hypothalamus
anterior
middle
posterior
Mc4r
female
male
Mrap2
female
male
PPa
PMp
v3
HD
HV
LH
B
Antisense Mc4r
Antisense Mrap2
OT
PGZ
TI
vm
v3
NDTL
HD
HV

Figure S2

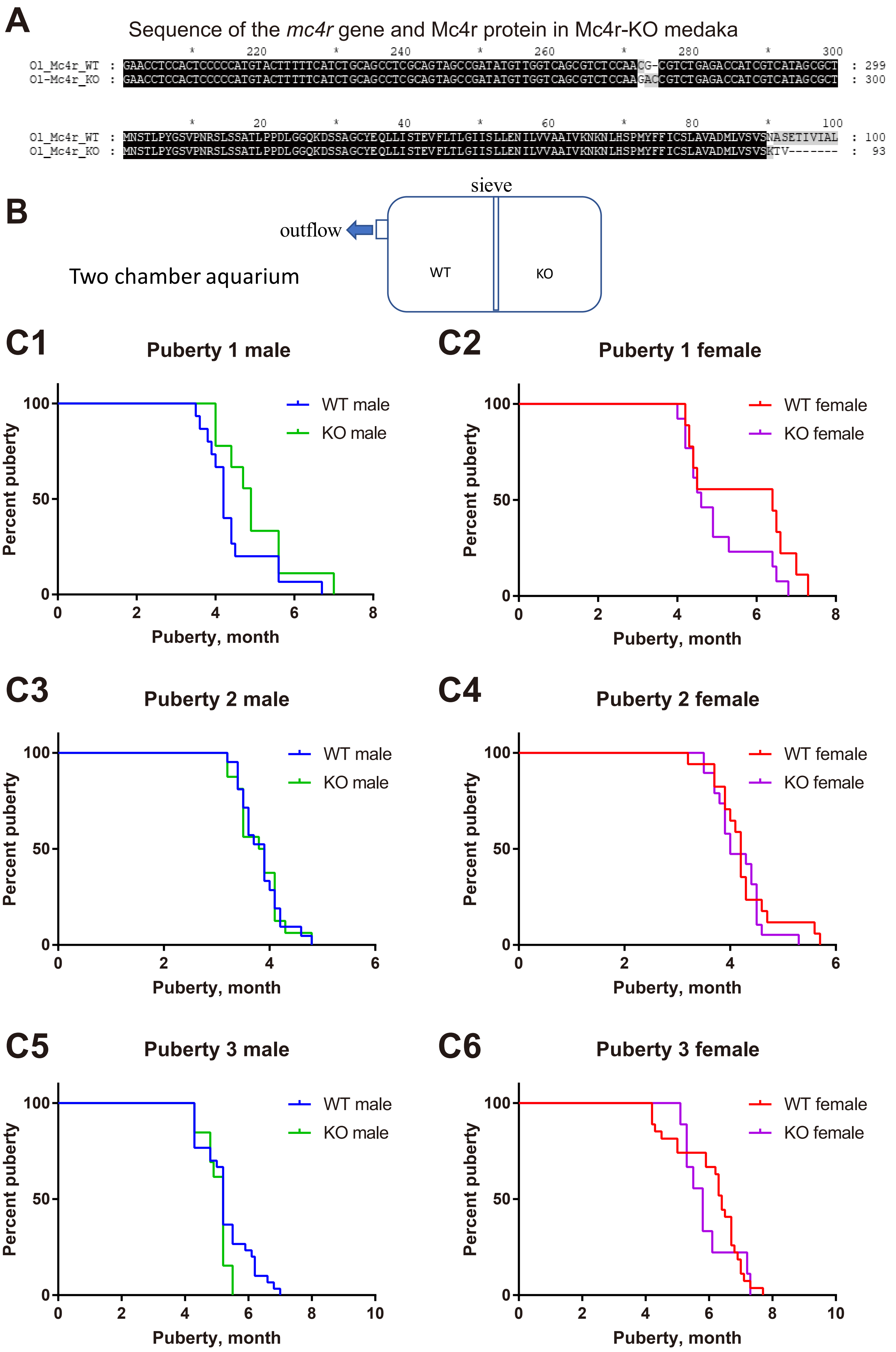
A
Sequence of the mc4r gene and Mc4r protein in Mc4r-KO medaka
* 220 * 240 * 260 * 280 * 300
Ol_Mc4r_WT : GAACCTCCACTCCCCCATGTACTTTTTCATCTGCAGCCTCGCAGTAGCCGATATGTTGGTCAGCGTCTCCAACG-CGTCTGAGACCATCGTCATAGCGCT : 299
Ol-Mc4r_KO : GAACCTCCACTCCCCCATGTACTTTTTCATCTGCAGCCTCGCAGTAGCCGATATGTTGGTCAGCGTCTCCAAGACCGTCTGAGACCATCGTCATAGCGCT : 300
* 20 * 40 * 60 * 80 * 100
Ol_Mc4r_WT : MNSTLPYGSVPNRSLSSATLPPDLGGQKDSSAGCYEQLLISTEVFLTLGIISLLENILVVAAIVKNKNLHSPMYFFICSLAVADMLVSVSNASETIVIAL : 100
Ol_Mc4r_KO : MNSTLPYGSVPNRSLSSATLPPDLGGQKDSSAGCYEQLLISTEVFLTLGIISLLENILVVAAIVKNKNLHSPMYFFICSLAVADMLVSVSKTV------- : 93
B
Two chamber aquarium
sieve
outflow
WT
KO
C1
Puberty 1 male
WT male
KO male
C2
Puberty 1 female
WT female
KO female
C3
Puberty 2 male
WT male
KO male
C4
Puberty 2 female
WT female
KO female
C5
Puberty 3 male
WT male
KO male
C6
Puberty 3 female
WT female
KO female
Percent puberty
Puberty, month

Figure S3

Supplementary Tables

Table S1. List of accession numbers of sequences of various species used in the study. All sequences are obtained from Ensembl Database or NCBI.

Mc4r

Gene	Specie	bp	Protein	Exon	Transcript ID	Protein ID
Mc4r	Amazon molly	990	329aa	1	ENSPFOT00000021127	ENSPFOP00000021099.2
Mc4r	Cave fish	1008	335aa	1	ENSAMXT00000027076	ENSAMXP00000027055.1
Mc4r	Coelacanth	990	329aa	1	ENSLACT00000000489	ENSLACP00000000487.1
Mc4r	Elephant shark	1017	338aa	2	XM_007895520.1	XP_007893711.1
Mc4r	Fugu	969	322aa	1	NM_001032560.1	NP_001027732.1
MC4R	Human	1666	332aa	1	ENST00000299766	ENSP00000299766.3
McrB	Lamprey	1035	344aa	1	BK007095.1	DAA34034.1
Mc4r	Medaka	1941	321aa	1	XM_004081195.4	XP_004081243.1
Mc4r	Southern Platyfish	963	320aa	1	KF650657.1	AHC02892.1
Mc4r	Spotted gar	5875	333aa	1	XM_015354632.1	XP_015210118.1
Mc4r	Stickleback	981	326aa	1	ENSGACT00000007378	ENSGACP00000007360.1
Mc4r	Tetraodon	972	323aa	1	ENSTNIT00000011720	ENSTNIP00000011538.1
Mc4r	Tilapia	984	327aa	1	ENSONIT00000025784	ENSONIP00000025763.1
Mc4r	Zebrafish	1084	326aa	1	ENSDART00000019555	ENSDARP00000027547.2
MC5R	Human	1319	325aa	1	NM_005913.2	NP_005904.1
Mc5r	Fugu	1023	340aa	4	NM_001032765.1	NP_001027937.1
Mc5r	Spotted gar	2852	328aa	2	XM_015357781.1	XP_015213267.1
Mc5r	Coelacanth	981	326aa	1	XM_005996516.1	XP_005996578.1

Mrap2

Gene	Specie	bp	Protein	Exon	Transcript ID	Protein ID
Mrap2	Amazon molly	2390	255aa	3	ENSPFOT00000019455	ENSPFOP00000019433.2
Mrap2	Cave fish	1511	218aa	3	XM_007237278.3	XP_007237340.2

Mrap2	Elephant shark	627	208aa	3	XM_007908433.1	XP_007906624.1
Mrap2	Fugu	687	228aa	3	XM_003971866.1	XP_003971915.1
MRAP2	Human	2153	205aa	4	ENST00000257776	ENSP00000257776.4
Mrap2	Medaka	2625	224aa	4	XM_020714712.2	XP_020570371.1
Mrap2	Southern Platyfish	1954	226aa	3	ENSXMAT00000013733	ENSXMAP00000013717.1
Mrap2	Spotted gar	1339	224aa	4	XM_006626027.2	XP_006626090.2
Mrap2	Stickleback	642	213aa	4	ENSGACT00000012548	ENSGACP00000012524.1
Mrap2	Tilapia	1725	264aa	4	ENSONIT00000018512	ENSONIP00000018495.1
Mrap2a	Zebrafish	1950	217aa	3	ENSDART00000148801	ENSDARP00000124181.1
Mrap2b	Zebrafish	600	199aa	3	ENSDART00000168266	ENSDARP00000132698.1
MRAP	Human	1023	172aa	5	NM_178817.3	NP_848932.1
Mrap1	Amazon molly	1359	124aa	5	XM_007568619.2	XP_007568681.1
Mrap1	Zebrafish	555	108aa	3	ENSDART00000148193.3	ENSDARP00000123129.2
Mrap1	Elephant shark	805	153aa	3	XM_007903550.1	XP_007901741.1
Mrap2a	Common carp	1949	214aa	3	XM_019119373.1	XP_018974918.1
Mrap2b	Common carp	615	204aa	3	XM_019086530.1	XP_018942075.1

Pomc

Gene	Specie	bp	Protein	Exon	Transcript ID	Protein ID
Pomca-1	Amazon molly	2246	249aa	3	XM_007569070.2	XP_007569132.2
Pomca-2	Amazon molly	794	183aa	3	XM_016681012.1	XP_016536498.1
Pomcb	Amazon molly	1025	236aa	3	ENSPFOT00000003135	ENSPFOP00000003131.1
Pomca	Cave fish	1003	221aa	3	XM_007244678.2	XP_007244740.2
Pomcb	Cave fish	878	197aa	3	XM_015607051.2	XP_015462537.2

Pomc	Coelacanth	768	255aa	2	ENSLACT00000013386	ENSLACP00000013290.1
Pomc	Elephant shark	1140	341aa	2	XM_007911414.1	XP_007909605.1
Pomca	Fugu	657	218aa	2	XM_011606335.1	XP_011604637.1
Pomcb	Fugu	2879	218aa	3	XM_003971727.2	XP_003971776.1
POMC	Human	1426	267aa	3	ENST00000405623	ENSP00000384092.1
Poc	Lamprey	1025	278aa	2	D55628.1	BAA09491.1
Pom	Lamprey	2045	245aa	2	D55629.1	BAA09492.1
Pomca-1 (Pomc-like)	Medaka	2350	265aa	3	XM_023959757.1	XP_023815525.1
Pomca-2	Medaka	898	212aa	3	XM_004066456.3	XP_004066504.1
Pomcb	Medaka	684	227aa	2	ENSORLT00000018657	ENSORLP00000018656.1
Pomca-1	Southern Platyfish	713	209aa	2	ENSXMAT00000001214	ENSXMAP00000001210.1
Pomca-2	Southern Platyfish	1442	257aa	3	ENSXMAT00000006432	ENSXMAP00000006424.1
Pomcb	Southern Platyfish	1408	236aa	3	ENSXMAT00000003692	ENSXMAP00000003687.1
Pomc	Spotted gar	1431	266aa	3	ENSLOCT00000020647	ENSLOCP00000020612.1
Pomca-1	Tilapia	1969	216aa	3	ENSONIT00000009130	ENSONIP00000009125.1
Pomca-2	Tilapia	814	208aa	2	ENSONIT00000023610	ENSONIP00000023589.1
Pomcb	Tilapia	1537	280aa	3	XM_005454720.3	XP_005454777.1
Pomca	Zebrafish	1224	222aa	3	ENSDART00000063333	ENSDARP00000063332.5
Pomcb	Zebrafish	648	215aa	2	ENSDART00000100751	ENSDARP00000091524.2

Agrp

Gene	Specie	bp	Protein	Exon	Transcript ID	Protein ID
Agrp1	Amazon	767	141aa	3	ENSPFOT0000000	ENSPFOP000000024

	molly				2445	41.1
Agrp1	Cave fish	1465	143aa	5	XM_022667066.1	XP_022522787.1
Agrp1	Cod	1364	128aa	3	BR000938.1	FAA00762.1
Agrp1	Elephant shark	588	155aa	4	XM_007889599.1	XP_007887790.1
Agrp1	Fugu	529	135aa	3	ENSTRUT00000009422	ENSTRUP00000009367.1
Agrp2	Fugu	907	115aa	3	ENSTRUT00000009410	ENSTRUP00000009355.1
AGRP	Human	764	132aa	4	ENST00000290953	ENSP00000290953.2
Agrp1	Medaka	3293	141aa	3	XM_011487064.3	XP_011485366.1
Agrp2	Medaka	1737	114aa	3	XM_004078892.4	XP_004078940.1
Agrp1	Southern Platyfish	578	140aa	3	ENSXMAT00000009960	ENSXMAP00000009946.1
Agrp1	Spotted gar	769	152aa	4	XM_006641519.2	XP_006641582.2
Agrp2	Spotted gar	3439	130aa	4	ENSLOCT00000012669	ENSLOCP00000012645.1
Agrp1	Stickleback	1130	132aa	3	BR000932.1	FAA00758.1
Agrp2	Stickleback	3507	115aa	3	BR000927.1	FAA00754.1
Agrp1	Tetraodon	393	130aa	3	ENSTNIT00000013316	ENSTNIP00000013124.1
Agrp1	Tilapia	429	142aa	3	ENSONIT00000004065	ENSONIP00000004064.1
Agrp1	Zebrafish	676	126aa	4	ENSDART00000135250	ENSDARP00000116390.1

Table S2. Primers used in this study.
Primers

Primers	Sequences	Comments
MF_ef1a1-f01	GCCCCTGGACACAGAGACTTCATCA	RT-qPCR for Elf1a
MF_ef1a1-r01	AAGGGGGCTCGGTGGAGTCCAT	RT-qPCR for Elf1a
Mc4r_Ol_F	GGCAACCTGAGCATTCCTGTCA	RT-qPCR for Mc4r
Mc4r_Ol_R	ATGTAGCGGTCAACGGCAATGG	RT-qPCR for Mc4r
MRAP2a_Ol_F	CGCACGACGCAGTGACTATGT	RT-qPCR for Mrap2
MRAP2a_Ol_R	ACCGCCAGTCCAACCCAGAA	RT-qPCR for Mrap2
Pomca_Ol_F	TGGACTCTGAGAGCAGCATGAC	RT-qPCR for Pomca
Pomca_Ol_R	AAGGGATCTGAGGGAGGTGGAG	RT-qPCR for Pomca
Pomcb_Ol_F	TTGCTGGCTGTTGGTGGTTCT	RT-qPCR for Pomcb
Pomcb_Ol_R	AGGTCTGGGCTTTCAGGTTTGA	RT-qPCR for Pomcb
AgRP_Ol_F	CATCCCTCACCAGCAGTCCT	RT-qPCR for Agrp1
AgRP_Ol_R	GCGGCAGTAACAGATGGCATT	RT-qPCR for Agrp1
Mc4r_Ol_F2	CCTGGGAGGACAGAAAGA	PCR for Mc4r *in situ* hybridization probe synthesis
Mc4r_Ol_R2	ATGAAGAGGATACCCGACA	PCR for Mc4r *in situ* hybridization probe synthesis
MRAP2a_Ol_F2	ACGAGTATTATGACGACGAG	PCR for Mrap2 *in situ* hybridization probe synthesis
MRAP2a_Ol_R2	GATGGTGTATCCCTGCTT	PCR for Mrap2 *in situ* hybridization probe synthesis

Table S3. Chromosomal location of Mc4r signaling system genes. Linkage group: LG.

	Southern platyfish	medaka
sex chromosome	LG21	LG1
mc4r	LG21	LG 20
mrap2	LG15	LG24
pomca1	LG13	LG11
pomca2	LG24	LG2
pomcb	LG15	LG24
agrp1	LG4	LG3
agrp2	LG6 (*asip2b*)	LG17

Table S4. Medaka developmental stages.
Days post fertilization: dpf; stage: S.

Days	Number of eggs in each pool	Stages
0dpf	100	S10-11
1dpf	50	S21-22
2dpf		S26
3dpf		S30
4dpf		S33
5dpf	30	S35
6dpf		S37
8dpf		8dpf Hatch
10dpf	15	10dpf
15dpf		15dpf
20dpf		20dpf

Supplementary sequences

Mc4r sequences in wild-type medaka.

>Ol_Mc4r_WT

ATGAACTCCACTCTGCCTTATGGGTCGGTCCCCAACAGAAGCCTCTCCTCGGCCACT
CTCCCTCCTGACCTGGGAGGACAGAAAGACTCGTCGGCGGGATGCTACGAGCAGCTT
CTGATCTCCACTGAGGTCTTCCTCACTTTGGGCATCATCAGCCTGCTGGAGAACATC
CTGGTTGTTGCTGCGATCGTTAAAAACAAGAACCTCCACTCCCCCATGTACTTTTTC
ATCTGCAGCCTCGCAGTAGCCGATATGTTGGTCAGCGTCTCCAACGCGTCTGAGACC
ATCGTCATAGCGCTCATTAACGGAGGCAACCTGAGCATTCCTGTCAGGCTCATCAAG
AGCATGGACAATGTGTTTGACTCCATGATCTGCAGCTCTCTGCTGGCCTCCATCTGC
AGCTTGCTGGCCATTGCCGTTGACCGCTACATCACCATCTTCTACGCTCTGCGATAC
CACAACATCGTGACGCTGCGGCGAGCAGCCGTGGTCATCAGCAGCATCTGGACGTGC
TGCATTGTGTCGGGTATCCTCTTCATCATCTACTCGGAGAGTACCACGGTGCTCATC
TGTCTCATCACCATGTTCTTCACCATGCTGGTGCTCATGGCCTCCCTCTATGTCCAC
ATGTTCCTGCTGGCACGTCTGCACATGAAGCGGATCGCGGCGCTGCCGGGCAACGCG
CCCATCCACCAGCGGGCGAACATGAAGGGCGCCATCACCCTCACCATCCTCCTCGGG
GTGTTTGTGGTGTGCTGGGCGCCGTTCTTCCTCCACCTCATCCTCATGATCACCTGC
CCCAGGAACCCTTACTGCACCTGCTTCATGTCGCACTTCAACATGTACCTCATTCTC
ATCATGTGCAACTCCGTCATCGACCCCATCATCTACGCTTTCCGGAGCCAGGAGATG
AGGAAAACCTTCAAGGAGATCTTCTGCTGCTCCAACGCTCTCCTGTGTGTGTGA

>Ol_Mc4r_WT

MNSTLPYGSVPNRSLSSATLPPDLGGQKDSSAGCYEQLLISTEVFLTLGIISLLENI
LVVAAIVKNKNLHSPMYFFICSLAVADMLVSVSNASETIVIALINGGNLSIPVRLIK
SMDNVFDSMICSSLLASICSLLAIAVDRYITIFYALRYHNIVTLRRAAVVISSIWTC
CIVSGILFIIYSESTTVLICLITMFFTMLVLMASLYVHMFLLARLHMKRIAALPGNA
PIHQRANMKGAITLTILLGVFVVCWAPFFLHLILMITCPRNPYCTCFMSHFNMYLIL
IMCNSVIDPIIYAFRSQEMRKTFKEIFCCSNALLCV

Mc4r sequences in -2/+3 TALEN-knockout medaka.

>Ol-Mc4r_KO-2/+3

ATGAACTCCACTCTGCCTTATGGGTCGGTCCCCAACAGAAGCCTCTCCTCGGCCACT
CTCCCTCCTGACCTGGGAGGACAGAAAGACTCGTCGGCGGGATGCTACGAGCAGCTT
CTGATCTCCACTGAGGTCTTCCTCACTTTGGGCATCATCAGCCTGCTGGAGAACATC
CTGGTTGTTGCTGCGATCGTTAAAAACAAGAACCTCCACTCCCCCATGTACTTTTTC
ATCTGCAGCCTCGCAGTAGCCGATATGTTGGTCAGCGTCTCCAAGACCGTCTGAGAC
CATCGTCATAGCGCTCATTAACGGAGGCAACCTGAGCATTCCTGTCAGGCTCATCAA
GAGCATGGACAATGTGTTTGACTCCATGATCTGCAGCTCTCTGCTGGCCTCCATCTG
CAGCTTGCTGGCCATTGCCGTTGACCGCTACATCACCATCTTCTACGCTCTGCGATA
CCACAACATCGTGACGCTGCGGCGAGCAGCCGTGGTCATCAGCAGCATCTGGACGTG
CTGCATTGTGTCGGGTATCCTCTTCATCATCTACTCGGAGAGTACCACGGTGCTCAT
CTGTCTCATCACCATGTTCTTCACCATGCTGGTGCTCATGGCCTCCCTCTATGTCCA

CATGTTCCTGCTGGCACGTCTGCACATGAAGCGGATCGCGGCGCTGCCGGGCAACGC
GCCCATCCACCAGCGGGCGAACATGAAGGGCGCCATCACCCTCACCATCCTCCTCGG
GGTGTTTGTGGTGTGCTGGGCGCCGTTCTTCCTCCACCTCATCCTCATGATCACCTG
CCCCAGGAACCCTTACTGCACCTGCTTCATGTCGCACTTCAACATGTACCTCATTCT
CATCATGTGCAACTCCGTCATCGACCCCATCATCTACGCTTTCCGGAGCCAGGAGAT
GAGGAAAACCTTCAAGGAGATCTTCTGCTGCTCCAACGCTCTCCTGTGTGTGTGA

>Ol_Mc4r_KO-2/+3

MNSTLPYGSVPNRSLSSATLPPDLGGQKDSSAGCYEQLLISTEVFLTLGIISLLENI
LVVAAIVKNKNLHSPMYFFICSLAVADMLVSVSKTV
